# Improvement Strategies for the Challenging Collaboration of General Practitioners and Specialists for Patients with Complex Chronic Conditions: A Scoping Review

**DOI:** 10.5334/ijic.5970

**Published:** 2022-08-08

**Authors:** Rebecca Tomaschek, Patricia Lampart, Anke Scheel-Sailer, Armin Gemperli, Christoph Merlo, Stefan Essig

**Affiliations:** 1 Center for Primary and Community Care, Department of Health Sciences and Medicine, University of Lucerne, Frohburgstrasse 3, 6002 Lucerne, CH; 2 Swiss Paraplegic Centre, Guido A. Zäch Str. 1, 6207 Nottwil, CH; 3 Swiss Paraplegic Research, Guido A. Zäch Str. 4, 6207 Nottwil, CH

**Keywords:** complex chronic disease, delivery of health care, integrated, primary health care, specialised care, interorganisational collaboration

## Abstract

**Introduction:**

Coordination of healthcare professionals seems to be particularly important for patients with complex chronic disease, as they present a challenging interplay of conditions and symptoms. As one solution, to counteract or prevent this, improving collaboration between general practitioners (GPs) and specialists has been the aim of studies by linking or coordinating their services along the continuum of care. This scoping review summarises role distributions and components of this collaboration that have potential for improvement for the care of patients with complex chronic conditions.

**Methods:**

Scoping review as a knowledge synthesis for components of collaboration and role distributions between medical specialists and GPs in intervention studies. The PubMed database was searched for literature from 2010–2020.

**Results:**

Literature search and reference screening generated 2,174 articles. 30 articles originating from 22 unique projects were included in our synthesis. In the interventions to improve collaboration, the GP is most commonly in charge of patient management and extends the scope of practice. The specialist provides support when needed. Clear definition of roles, resources for knowledge transfer and education from specialists are commonly utilised interventions. Typically, combinations of process and system changes addressing communication and coordination issues are applied. Most interventions improve provider and patient satisfaction, health outcomes, and reduce care fragmentation.

**Conclusion:**

This review showed that interventions to improve collaboration between GPs and medical specialists seem promising. Further efforts should be made to test and apply the findings systematically in broad clinical practice.

## Introduction

A lack of coordination between care levels has been identified as one of the major challenges in current care provision. It is known that a lack of coordination results in duplicate provision of healthcare services, inconsistent medical advice and poor patient satisfaction [1]. To tackle the effects of missing coordination, multiple strategies and frameworks have been developed and applied [2]. Approaches to link levels of care to improve coordination have been developed and are referred to as integration of care. Vertical integration of care, on the one hand, is based on a disease-focused perspective that is organised around a provider-based silo. Horizontal integration of care, on the other hand, has a holistic, person-focused perspective [3]. For both approaches, interprofessional partnerships are needed that deliver comprehensive care for a defined population based on shared competences, roles and responsibility [3,4]. To support building interprofessional partnerships, interprofessional education programs were introduced. These approaches, based on interprofessional team building for comprehensive and continuous care have shown to be effective [5,6]. However, progress and acceptance of these teams seem to differ between countries and healthcare systems [7].

One major element to achieve integrated care is a general practitioner (GP) as a first point of contact for patients and for navigating the way through the healthcare system and its institutions [2,3]. Especially in rural areas, GPs are the first and only point of contact for patients and provide access to services in collaboration with other healthcare professionals (HCPs) [8]. Collaboration refers to “a process of problem-solving, shared decision-making and to carry out a care plan while working towards a common goal” [5, p.133]. However, this collaboration inherits local, institutional and healthcare system barriers that need to be overcome. Common challenges are a lack of clearly distributed responsibilities between professionals and absence of mechanisms to communicate or exchange information [8,9,10,11]. Nevertheless, GPs interact with a large number of medical specialists to have access to their specialised knowledge.

Coordination of healthcare professionals seems to be particularly important for patients with complex chronic disease, as they present a challenging interplay of conditions and symptoms [2,12,13]. Due to their manifold medical, social and behavioural factors, patients typically require the attention of multiple healthcare providers, facilities or home-based care [14]. Especially, the absence of a commonly shared information system complicates information exchange; oversight and comprehensive knowledge on the patients’ situation is lost [15,16]. Therefore, patients with complex chronic conditions are more vulnerable to fragmentation of care [17]. As one solution, to counteract or prevent this, improving collaboration between GPs and specialists has been the aim of studies by linking or coordinating their services along the continuum of care [18]. Successful collaborative care approaches make the specialist’s expertise and the GP’s comprehensive view available to patients [9]. Enhancing the collaboration between care providers was able to eliminate competition and unequal access to services [19]. Both, GPs and specialists, are expected to care for patients with chronic conditions and are consulted for their disease management skills. The roles of GPs and specialists overlap but vary regarding the degree of focus on specific organs. Therefore, specialists are visited sporadically but usually do not continuously manage stable conditions [17,20].

Although research provided approaches to improve collaboration aiming to reduce fragmentation of care, implementation of these approaches in a broader context appear to be slow. Furthermore, it seems that results from some newly introduced, collaborative models are not universally applicable, because they do not reflect the prevalent problem of multimorbid patients and focus on one chronic condition [2,21]. Some researchers fear that “disease-specific organisation of care delivery based on guidelines potentially limits the possibilities for tailoring care delivery to individual patients” [21, p.26]. In addition to this challenge, the central components of collaboration are not evident yet even though these studies have shown that collaboration impacts patient outcomes positively. To establish a best practice to achieve integrated care, these components need to be summarised [2,12].

This scoping review provides an overview of intervention studies that aimed to improve collaboration between GPs and specialists for patients with complex chronic disease. We summarise components of collaboration, as well as role distributions. Ultimately, we want to provide guidance on how to improve the collaboration between GPs and specialists.

## Methods

The review aims to answer the research question: How can GPs and specialists improve their collaboration to improve care for patients with complex chronic conditions? To answer this question, we identified the components of collaboration that were addressed in intervention studies. Furthermore, we summarised the role distribution between GPs and specialists that was developed within the interventions. Lastly, we summarised how interventions were evaluated and what outcome measures were assessed.

We chose a scoping review as a knowledge synthesis methodology due to the studies’ broad research approaches. These approaches include a range of different outcome measures and analytical frameworks. Furthermore, we did not consider the quality of the studies as suggested for scoping reviews [22]. The research question was phrased according to the PICO format (supporting table 1) [23]. The review follows the recommendations of the PRISMA-ScR checklist [24].

### Literature search

The search strategy consisted of a combination of free text and Medical Subject Headings (MeSH) with Boolean Operators and was structured according to four general concepts: 1) patient population 2) primary care physicians 3) specialised care physicians and 4) type of care provided to the patient. Four previously known articles were used to test whether the search strategy was able to identify suitable literature. Search terms were added until all four articles were identified. Finally, studies were searched in the PubMed database. The final search string is presented in the supporting table 2. Last, we identified further relevant literature from the reference lists of selected articles.

### Inclusion criteria

Original peer-reviewed intervention protocols and studies with a quantitative design published between 2010 and 2020 written in any language were eligible for inclusion in this review. Publications were included if 1) the intervention aimed to improve collaboration between GPs and medical specialists and 2a) they provided information on components of the interface for shared care and collaboration, or 2b) defined role distributions between medical specialists and GPs. Articles were excluded if 1) the design was not prospective with either a control group or before-and-after comparison or 2) the intervention involved only other HCPs besides physicians to improve healthcare provision.

### Selection

We extracted potentially eligible literature from the database and checked for inclusion in two steps. First, titles and abstracts were checked if primary and secondary care physicians worked together. Second, full texts were read to ensure that the study sufficiently reported on the collaboration model, as well as the development process and concepts behind it. Two researchers (PL, RT) performed the study selection independently. We discussed studies with a third reviewer (SE) when the selection decision differed. The decision to include was based on consensus among all three researchers.

### Data extraction

Information that contributed to answering the review’s research question was extracted into a spreadsheet. The sheet synthesises key information on each paper (supporting table 3). This process was carried out by one researcher (RT). RT and SE regularly discussed the spreadsheet to ensure that it provides on the necessary information.

## Results

**Figure 1:**
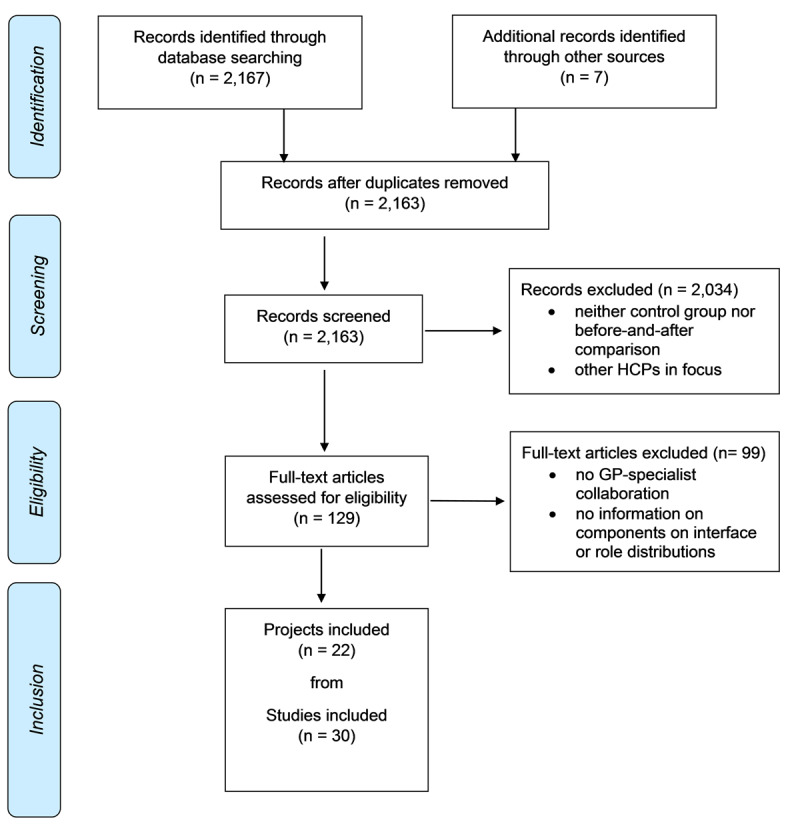
Preferred Reporting Items for Systematic Reviews and Meta-Analyses” (PRISMA) flow diagram of the study selection process [25].

As shown in Figure 1, the literature search generated 2,167 potentially eligible publications and further reference screening yielded 7 additional publications. After duplicate removal, title and abstract screening, 129 full texts were assessed for eligibility. We found multiple articles reporting on the same project. Finally, 30 articles from 22 projects were included in this review. The results are presented as 22 unique projects.

**Figure 2:**
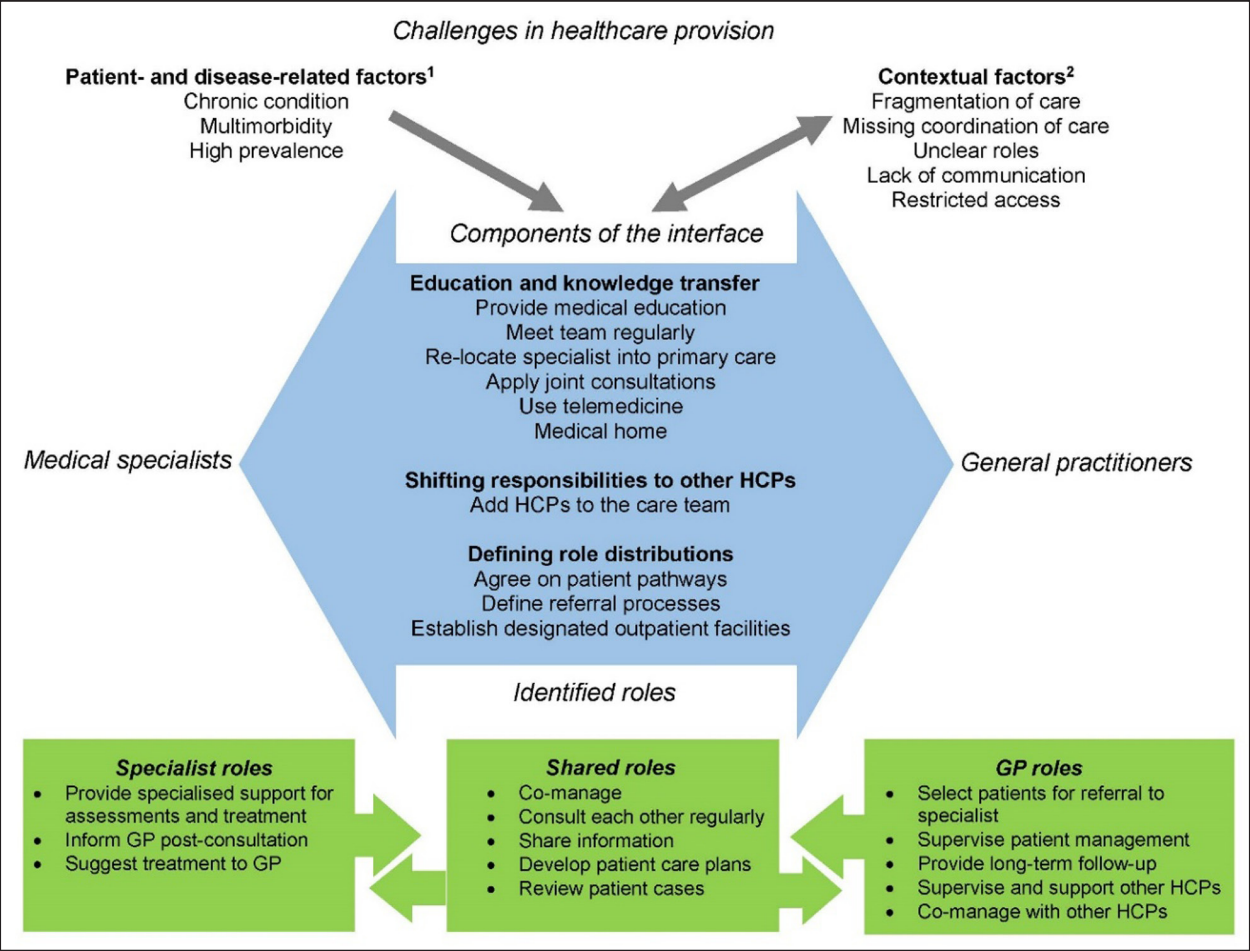
Overview of challenges in healthcare provision, components of the interface of collaboration and role distributions as identified from the literature. HCPs: healthcare professionals; GP: General practitioner. 1: Patient- and disease-related factors refer to challenges in healthcare provision that stem from patient characteristics or characteristics of their conditions. These factors influence the collaboration between specialists and general practitioners, but cannot be modified by the collaboration. 2: Contextual factors originate both from the healthcare system and the collaboration at the primary and secondary care interface, i.e., these factors influence the collaboration between specialists and general practitioners, but can also be modified by this collaboration.

Figure 2 summarises the review`s results. It visualises the challenges that influenced the design of interventions (chapter 3.1.1), the main components of collaboration that were implemented in interventions (chapter 3.2) and the role distributions resulting from the interventions (chapter 3.3).

### Project characteristics

#### Rationale for projects: Challenges in healthcare

Authors designed their intervention with challenges in the healthcare provision in mind. These challenges were either related to patient and disease-related factors or contextual factors. Fragmented care, a barrier to care provision, was one contextual factor that was focused on in interventions. More specifically, reasons for fragmented care were reported to be a lack of communication and cooperation in care that was vulnerable to miss patients at risk. It ultimately resulted in the delayed provision of care [26,27]. The lack of clear roles of involved HCPs, guidelines and referral processes limited the patients’ access to care [28,29,30,31]. Lack of knowledge and awareness of GPs on the condition resulted in a shortcoming of care and preventive measures [32,33,34]. The current organisation of care and its coordination was often considered suboptimal or even outdated for the specific health condition. As part of patient- and disease-related factors, authors specified that highly prevalent health conditions were focused on in the interventions. Authors reported on the respective condition’s burden on healthcare facilities or system resources such as high consultation rates [30,31,35] or costs [36,37,38,39,40,41]. Furthermore, projects presented that the health condition of interest is a leading cause of morbidity, hospitalisations and death [37,38,39,40,42,43,44,45,46]. Additionally, the conditions’ complexity was mentioned. Interestingly, only few projects reported on what they defined as complex patient care or complex needs even though most of them used this term to describe patient-related characteristics. These definitions included: presence of polypharmacy [30,31], a minimum amount of follow-ups [36], presence of multiple interventions to provide stage- and symptom-dependent care [34], presence of multimorbidity [41,47], presence of multiple practitioners [48] and patients’ “ongoing management of their [condition] is beyond the capability of their usual GP” [49, p.1113].

#### Origin and study design

Of the 22 original projects,

- five were from the United States [26,30,31,38,39,40,42,43,44,50,51],
- four were from Canada [28,29,36,37,48];
- two projects were from Australia [49,52,53], Brazil [47,51], Spain [35,54], Switzerland [33,34,45] and the Netherlands [27,55].
- and one project was from Germany [32], Italy [41] and New Zealand [46], respectively.

Eight projects applied a before-and-after design with outcome measurements pre and post intervention [26,30,31,37,38,39,40,41,47,50], whereof three before-and-after designs further compared the intervention group to a control group [45,53,54]. Additionally, three projects compared the intervention group to a control group, which was usual care [35,48,49,52]. Five projects were RCTs [27,33,34,42,43,44,46,55]. One project used an array of methods for their evaluation; the authors applied a randomised stepped-wedge design, a before-and-after and a controlled design separately [28,29]. Furthermore, two protocols for RCTs [36,51] and one protocol for a before-and-after design [32] were included in this review.

Seven projects applied randomisation, and of those, two projects randomised on a patient level [28,29,42,43,44] and five on a healthcare provider level (e.g. long-term care home, GP practice) [27,33,34,36,46,55]. Three projects blinded the involved HCPs with regard to the allocation sequence [28,29,42,43,44,46] one mentioned specifically that blinding was not possible [28,29] and three gave no information whether any study participants were blinded [27,33,34,51,55].

Twelve projects reported an intervention period of 12 months [27,28,29,33,34,36,37,45,48,49,50,51,52,53,55] and one project of 10 months [30,31]. Nine projects evaluated the intervention for more than one year with a mean duration of approximately 40 months, ranging from 15 to 156 months [26,32,35,38,39,40,41,46,47,54].

#### Patient population

Projects involved various patient populations with either specific or unspecific diagnoses. Table 1 summarises the diagnoses or descriptions of the patient populations’ characteristics.

**Table 1:** Patient population in interventions.


	PATIENT POPULATIONS’ DIAGNOSES	REFERENCES

Specific diagnoses	chronic kidney disease (CKD)	5 projects [26,27,37,38,39,40,41]

	chronic obstructive pulmonary disorder (COPD)	3 projects [33,34,45,50]

	diabetes type 2	2 projects [49,52,55]

	combination of asthma and COPD	1 project [47]

	chronic hepatitis B	1 project [53]

	chronic heart failure	1 project [42,43,44]

	coronary artery disease	1 project [51]

	at least one of the following was present: diabetes, cardiovascular disease, COPD, asthma or cardiovascular risk factors	1 project [28,29]

Unspecific patient population (regarding diagnoses)	long-term	2 projects [36,46]

	end of life care	1 project [32]

	multiple chronic conditions	1 project [30,31]

	heart diseases	1 project [54]

	gastroenterology and hepatology	1 project [35]

	kidney diseases	1 project [48]


### Organisation of care and components of collaboration

Interventions implemented process and system changes to enhance collaboration between primary and secondary care. The measures focused on education, as well as information exchange and knowledge transfer, as well as shifting responsibilities to additional HCPs.

#### Education, communication and information exchange

Formal education and training sessions for GPs and primary care staff was widely applied [32,35,46,49,52,54]. Adams et al. created a CME program that targeted primary care providers and aimed to improve their knowledge/comprehension, self-confidence, and, ultimately, clinical practice [50]. Pang et al. developed a mentor-mentee program with individual training sessions, regular regional learning meetings and case consultations. The intervention’s educational aspect focused on early detection and identification of patients with CKD [37]. A Dutch project focused on educating primary care staff in blood pressure management and treatment, cholesterol lowering, blood-glucose management, and lifestyle advice for patients with long-term hypertension or diabetes type 2 [55]. Specific education topics in other projects were early identification of an at-risk population, effective prevention and treatment strategies and inappropriate drug utilisation [33,34,41,47,53]. Fortin et al. arranged a chronic disease prevention program with a focus on patients’ self-management. Included HCPs received training in motivational interviewing to help reduce the burden of chronic diseases and related high-risk lifestyles [28,29].

Furthermore, GPs and primary care staff enhanced their knowledge in daily practice. Mostly, they were encouraged to communicate more and share information with each other and specialists. After receiving formal CME, Tinetti et al. encouraged physicians to review selected patient cases together and to identify patient priorities. The primary and secondary care physicians were stimulated to align their care towards patient priorities and the same outcomes [30,31]. Martins’ et al. study improved the physicians’ health condition specific knowledge before inviting a medical specialist to perform joint consultations in the GP practice [47]. Before opening specialised outpatient clinics, Santoro et al. provided teaching sessions. Within the outpatient facilities GPs were then asked to profile patients, assess their co-morbidities and renal insufficiency stage, plan lab tests and referrals. Furthermore, if appropriate, GPs developed personalised therapy and nutrition plans [41]. Some studies provided care arrangements between GPs and specialists that included hands-on teaching or experience exchange without planning formal education events [26,30,31,35,36,55]. As an example, Falces et al., integrated a cardiologist into a primary care practice to support follow-up [54]. Similarly, Hynes’ and Porter’s project introduced new HCPs in the patient-centred medical home, who shared experiences and information in multidisciplinary team meetings [38,39,40]. Besides multidisciplinary team meetings [42,43,44,45,46], measures to enhance communication included regular round table discussions [47], regional meetings [37] and web-based tools for simplified informal exchange [27,41,48,53].

To support formal information exchange, several interventions provided screening tools [26], guidelines [26,32,33,34], treatment protocols [53], standardised discharge processes [51] and defined referral procedures [26,33,34,35,48,53,54]. Other formal possibilities included a virtual co-management tool. It supported providers with guiding referral processes that visualised patients’ pathways [48]. Other electronic platforms were incorporated to store patients’ health records [27], exchange documents [48], and facilitate direct interaction [36,51]. Ho et al. focused on providing telemedicine consultation for GPs and provided educational documents on evidence-based care of geriatric patients [36,51]. Usually, these standards and tools were developed before the project and in accordance with project participants and experts to ensure applicability.

#### Shifting responsibilities to other HCPs

Additional HCPs besides physicians were widely integrated at the primary-secondary care interface. Nurse practitioners, nurse educators, specialised nurses, nurse coordinators and practice nurses were the professionals most frequently included in interventions. Rather passive involvement in the care provision included medical education of nurses, physician assistants, medical students, respiratory physiotherapists without any further specified or assigned roles [33,34,35,50,51]. As an active part of the intervention, HCPs took over organisation-related and patient-related responsibilities with different tasks.

Organisation-related tasks included:

- nurse coordinators or specialised nurses arranged multidisciplinary team meetings [46]
- projects’ research staff was performed pharmacological consultations for HCPs and medication reviews [36]

HCPs’ patient-related tasks were:

- community health worker and a pharmacist were added to the team in a long-term care home [38,39,40]
- social worker performed psychosocial assessments [42,43,44]
- pharmacists, physiotherapists and community health workers who provided patient education on profession-specific care [38,39,40,45]

### Role distributions

Some projects defined roles prior to the intervention and others openly gave physicians the task to define responsibilities themselves. The following synthesises the roles, how they were distributed among GPs and specialists or shared between them.

Many interventions assigned clearly distinct roles to GPs and specialists. The more severe the disease and symptoms, the more likely a specialist was involved in patient care. The GP was expected to support with problems of little to moderate severity. Accordingly, the patients were referred to the specialist for specialised assessments and treatments [30,31,32,33,34,41,53]. Furthermore, several projects expected specialists to provide medical education to GPs. GPs were expected to enhance their knowledge and services by receiving CME [30,31,33,34,37,49,52]. Pang et al. assigned GPs to specialists, acting as mentors. In brief, an assigned mentor was responsible for regular education sessions and offered one-on-one consultations, but little supervision during daily practice. In this project, the GPs knowledge on diagnosing and managing patients with CKD was evaluated [37]. Another project encouraged a hospital to distribute specialised patient care plans to GPs upon referral. Management was transferred to GPs but overseen with an electronic treatment protocol by the hospital team. The primary care team was evaluated regarding their ability to treat and monitor patients with CKD [53]. In a Swiss project, the GP was asked to follow a checklist closely and their adherence was monitored by the research team [33,34].

Other studies included interventions on joint consultations or patient case discussions. They allowed for more direct interaction between physicians [27,36,47,48,51]. For instance, van Gelder et al. integrated a web-based consultation tool to enable GPs to align their care with nephrologists by consulting them. The GP was responsible for patient care and data entry into patients’ electronic medical records. Specialists had access to the same information and provided streamlined advice [27]. Falces et al. integrated a specialist into the primary care team as a new member to assist in patients’ follow-up and to distribute the patients between care levels. The physicians were able to establish their own referral criteria and actively discuss their different responsibilities. In this case, specialists provided medical advice when needed, and had weekly consultation sessions with GPs [54]. Even though the specialist took the lead in joint consultations with the GP and patients in a Spanish study, the GP remained the main person of contact for the patient [35]. In the project of Fortin et al., GPs were in charge of patients’ long-term follow-up, while the specialist took a rather passive role and had regular contact with the GP for support accordingly [28,29]. Haley et al. motivated GPs and specialists to develop tools addressing specific barriers and root causes for problems related to collaboration. In this project, the GP and specialist were considered equally important for patient care and co-managed within their expertise’s scope. The GP was in charge of patient management and sought specialised support when needed [26].

In projects that integrated additional HCPs into primary care institutions, the GP was expected to supervise and to provide support. The GP was usually in charge and responsible for patient care but delegated certain tasks and responsibilities [38,39,40,42,43,44,45,49,52,55]. Compared to a traditional primary care setting, a project taking place in a long-term care home is co-managed by the GP and other HCPs without the GP taking the lead or being superordinate [46].

### Outcomes and results of projects

The majority of projects analysed patient-related outcomes. Overall, the results were positive and reported improvements in outcomes. Several projects reported on improvements in patients’ health condition-specific outcomes. They included HbA1c level [49,52], blood pressure [55] or estimated glomerular filtration rate [27,41]. Falces et al. declared that an improvement in body mass index, better control of blood pressure and cholesterol were achieved [54]. No change was reported in the primary outcome, a patient-reported disease-specific HRqoL questionnaire, but secondary outcomes (depression, fatigue and anxiety) improved [42,43,44]. Two projects evaluated referral rates to the specialist and found a decrease at project termination [35,47]. Connolly’s et al. measured a decrease in avoidable emergency department admissions [46].

Self-reported physician outcomes included improved clinician confidence [50], satisfaction [54], increased knowledge [37,50], heightened attention to communication and awareness for a health condition [26]. Furthermore, the GPs’ participation rate in one-on-one case discussions with specialists was reported [37]. Additionally, process-related outcomes included improved outcome documentation by the GP [26] and an improved disease-specific medication dispensing [47]. Finally, some interventions were reported to increase costs slightly [27] or to not increase resources [54].

## Discussion

### Main findings

This review provides a broad insight into interventions aiming to improve collaboration between GPs and medical specialists at the primary and secondary care interface. There was no gold standard on the measures and different process and system changes were implemented.

Knowledge transfer between physicians was a key factor in patient case discussions, joint consultations and medical education. Many interventions aimed at developing clear role distributions between physicians and showed that GPs are able to take on additional responsibilities successfully with streamlined specialist support. Ultimately, improving the GP-specialist collaboration increases provider and patient satisfaction, and health condition-specific outcomes.

Even though this scoping review focuses on the collaboration between GPs and specialists, many interventions were interprofessional. Due to increasingly complex patient needs, primary care surely needs a skill mix and improving the collaboration between GPs and specialists is one of many steps towards integration of care.

### Interpretation and comparison with existing literature

In line with qualitative research, the importance of clarifying roles to improve collaboration has been highlighted. Clear role descriptions supported to respect, trust and recognise each other’s expertise and a lack can be a significant barrier to collaboration and created power struggles [4,11]. The collaboration between GPs and specialists, can be classified as interorganisational; that is when healthcare professional representing different organisations engage for patient care. This type of collaboration seemed to need a more formal role clarification process [4,11]. We identified examples such as formal discussions or the development of tools to specify responsibilities and roles within the intervention [26,54]. With or without a formal process, the GPs role as a continuous point of contact for patients was emphasised in various healthcare conditions and settings. This finding was not surprising as the GPs’ role as a gatekeeper giving access to specialised healthcare is one essential integrative function of primary care [3]. Specialists were mostly employed as comanagers or consultants [26,28,29,35,54] or they were not asked to supervise GPs by e.g. checking guideline adherence [33,34,53]. According to Forrest, consultants can provide diagnostic or therapeutic advice to reduce uncertainty or perform services to aid diagnosis, cure a condition, palliate symptoms or identify and prevent conditions [20]. Both types of specialist consultants were present in the included studies.

Similar to our findings, the values of education and knowledge transfer have been documented in qualitative and observational studies. GPs reported to invest themselves in collaboration with specialists to extend their medical knowledge and ultimately their services and perform better patient care [13]. Furthermore, education events facilitated personal relationships between healthcare professionals. The quality of relationships with specialists seemed to be especially important for GPs and to have implications for patient care. Knowing each other personally appeared to support the establishment of informal networks with irregular professional contacts by GPs [4,8,13]. D’Amour et al. highlighted that the familiarisation process to get to know each other personally and professionally needs to occur at social occasions, as well as informal and formal exchange events. Only then, values and level of competences are sufficiently transparent to form common goals for patient care and trust [10]. Furthermore, competencies for collaborative care can be learned and enhanced during these events [9]. Accordingly, the included interventions based on education and knowledge transfer were rather quick to implement. Some studies incorporated formal CME programs [47,50] or combined education with knowledge transfer between physicians during daily practice [37,38,39,40,42,43,44,54]. Specific health-condition education focussed on early identification of an at-risk population, effective prevention and treatment strategies [33,34,41,47,53,55]. Care arrangements between GPs and specialists were usually based on hands-on teaching without planning formal education events. Some of these studies encouraged co-management of patients and the specialist was involved more, as soon as the GP needed assistance in the management [41,54]. Others studies offered physicians time to discuss patient cases together and exchange formal information [30,31,47].

We know very well from previous research that inoperative referrals and discharges are interrupting continuity of care. Especially, a lack in communication between care levels delayed follow-up treatments and might result in unnecessary re-admissions [16]. Therefore, theoretical frameworks suggest to improve connectivity [10,21]. “Connectivity allows for rapid and continuous adjustments in response to problems of coordination” [10, p.5]. As suggested by those frameworks, several interventional studies tried to improve discharge and referral process [26,45,48,51,54], as well as screening tools and guidelines [28,29,32,33,34,49,52,55].

As indicated by our findings and other studies, electronic tools can be implemented to connect physicians separated by space and time [20]. Indeed, electronic consultation tools were introduced in intervention studies to provide remote assistance for decision-making and support by specialists for GPs. Reasons for integrating e-consultation tools included improved specialist access and reductions in unnecessary in-person referrals [36,48]. Furthermore, the tools added diagnostic and therapeutic support as well as a way to provide continuing health education [51]. However, further conceptual frameworks suggest electronic tools as a measure to enhance not only coordination, but also communication, direct exchange of documents, educational material and discussions needs to be possible [20]. Several identified studies developed and implemented one tool that combined all or most of those aspects [27,36,48,53].

General concepts of interprofessional collaboration are not only an important background to the review [5], but they are also applicable in interventions. Many studies added more HCPs to the traditional physician collaboration and created interprofessional teams. Responsibilities and HCPs’ involvement in the patients’ care processes varied between projects. Nurses engaged rather actively and performed patient assessments, developed and reviewed care plans, coached staff, took over patient education and shared responsibilities with GPs [26,28,29,37,38,39,40,42,43,44,45,46,49,52,53,55]. Other HCPs, such as therapists and pharmacists, were expert consultants that provided specialised advice or services [38,39,40,45]. Interprofessional teams might otherwise be interpreted as a competition to the traditional GP-specialist collaboration, but forming teams is one important approach towards vertical integration of care [3].

### Implications for research and practice

This scoping review has identified several mostly successful projects to improve collaboration between GPs and specialists. Despite these findings, reduction of care fragmentation seems to be slow. The following aspects might contribute to slow implementation of research findings:

- More than half of the projects lasted twelve months or less, which might be rather short to analyse quality of healthcare provision or knowledge implementation. Longitudinal study designs are needed, as these types of interventions are related to transitional processes.
- To confirm research findings and ensure generalisability, the interventions should be replicated in other settings and tested in clinical practice.
- Some studies seemed to be designed without taking clinical practice into account. These studies should be designed with relevant stakeholders. For example, researchers, HCPs and policy makers should jointly decide on the choice of outcome measures.
- Lastly, as mentioned in the results synthesis, only few projects provided a definition of complexity. Researchers should be aware that this concept is not used consistently among the scientific community and providing an understanding on how the term was defined in their context is essential for reproducing.

All projects were able to identify challenges in collaboration that were addressed in the interventions accordingly. This might seem obvious, but stakeholders should be aware that interventions could be “overdesigned”. A thorough analysis of the situation should be performed to prevent interventions from losing focus trying to address too many issues. In fact, identified projects rarely created new care models and rather extended existing ones with additional measures. In particular, process-related changes to better manage and coordinate, seemed to be implemented easily and showed changes for clinicians and researchers quickly. Joint patient consultations, adding new HCPs with own responsibilities or the implementation of web-based tools/telemedicine as examples for system changes, needing a larger commitment from involved parties. Of course, the interventions’ generalisability is not given. Presented measures cannot simply be introduced into daily practice to achieve the same results, as contextual factors most likely differ. Therefore, it should be stressed again that an analysis of the collaboration prior to designing an intervention is essential.

### Limitations and strengths

We observed, that the reporting of interventions affected our result synthesis. Authors should consider that their reporting informs other researchers applying similar concepts, who highly benefit from a detailed process description for designing the intervention. Moreover, the settings and outcomes of projects vary widely and the mix is sometimes hard to interpret and compare. In particular, projects with GP-specialist collaboration as one part of a larger intervention, limit the possibility to determine the model parameters of interest. As an additional limitation, it is likely that relevant published articles were not identified as the review includes literature from only one database published between 2010 and 2020. We realise that there might be unpublished projects from medical societies or governments, such as introducing compulsory continuing professional development. However, the amount of published literature seems to be sufficient to provide an overview of interventions. Furthermore, the included study protocols’ comparability may be limited regarding projects that already reported results. These protocols were included, as we wanted to focus on concepts in a broader health system perspective, instead of summarising outcomes or effect sizes of interventions. Besides the listed limitations, this scoping review provides an informative overview for researchers, HCPs, policy makers and other stakeholders wishing to improve collaboration between specialists and GPs. The presented measures were shown to achieve this goal.

## Conclusion

This scoping review aimed to provide an overview of intervention studies trying to improve the collaboration between GPs and medical specialists. An array of collaboration components, as well as role distributions was identified. All interventions seemed to be newly designed for the respective setting with its facilitators and barriers. Common components to increase collaboration included providing medical education, enabling informal knowledge transfer and process changes related to coordination. Quite often, the latter was achieved by integrating formal tools such as checklists, guidelines and standardised protocols. Physicians defined role distributions themselves in some interventions which usually resulted in cooperative or shared care arrangements, where the GP took over more responsibilities.

This review showed that interventions to improve collaboration between GPs and medical specialists seem promising. Further efforts should be made to test and apply the findings systematically in broad clinical practice. Researchers should keep in mind to report their interventions thoroughly to ensure replicability and to implement interventions for a sufficient amount of time to report changes reliably.

## Data Accessibility Statement

All data generated or analysed during this study are included in this published article and its supplementary information files.

## Additional File

The additional file for this article can be found as follows:

10.5334/ijic.5970.s1 Supporting Tables. Tables 1 to 3.
