# Supplementary material for: Improvement Strategies for the Challenging Collaboration of General Practitioners and Specialists for Patients with Complex Chronic Conditions: A Scoping Review
Source: Int J Integr Care. 2022 Aug 8;22(3):4. doi: 10.5334/ijic.5970 (PMC9374013; doi:10.5334/ijic.5970)
Supplement: Supporting Tables. — Tables 1 to 3. [file ijic-22-3-5970-s1.zip › s1-ijic-5970_tomaschek/5970-25270-1-SP.docx]

*Supporting Table 3: Details of included studies*

| Study characteristics | | | Patient population | Healthcare institutions/ professionals | Intervention in | Outcomes and results |
| --- | --- | --- | --- | --- | --- | --- |
| First author and year: Adams et al., 2012 | Location: United States, urban, multiple sites | Design and intervention period: Before-and-after study, 12 months | N= none Health condition: COPD | N(GPs and specialists)= 74 N(physician assistants, nurse practitioners, pharmacists, registered nurses, respiratory therapist)= 277 | Organisation of care: Educational program developed by (1) linking instructional methods to outcome strategy, (2) creating teachable moments, (3) using formative assessment throughout the process, and (4) fostering true collaboration among the various disciplines. | Self-reported clinician self-confidence (++), self-reported knowledge/comprehension (+), self-reported clinical practice (+) |
|  |  |  |  |  | Role-distribution: GPs and other involved primary care personnel receive medical education by specialists. |  |
| First author and year: Afshar et al., 2019 | Location: Germany, region (urban and rural), multiple sites | Design and intervention period: Study protocol for before-and-after study, 36 months | N= not reported Health condition: oncological and/or non-oncological chronic progressive diseases (long-term, end of life) | N(GPs)= 50 N(palliative care provider incl. specialists)= 15 | Organisation of care: Provide GPs and practice staff with standardised training to improve care of patients with chronic progressive disease in the last phase of their life. | No outcomes yet. |
|  |  |  |  |  | Role-distribution: GPs treat symptoms and provide support in problems of little to moderate complexity in all four dimensions (physical, psychological, social and spiritual); communication, clarification of therapeutic objectives and coordination of care; Involvement of specialised primary care when indicated. Specialists develop guidelines and supervise. |  |
| First author and year: Askew et al., 2010 / Russel et al., 2013 | Location: Australia, urban, one site | Design and intervention period: Controlled study, 12 months | N= 328 Health condition: Complex diabetes type 2 (long-term) | N(HCPs)= not reported | Organisation of care: Case-based education sessions for referrals GPs by specialists and employment of GP clinical fellows with advanced training in diabetes care. Development of a management plan and regular follow-up visits of the patient. | Disease-specific patient outcomes (+) Clinical safety, quality and acceptability of care model are demonstrated. |
|  |  |  |  |  | Role-distribution: Specialists develop and provide the training program for GPs and review the patient management plan. GP provides patient care within the new care model. Diabetes nurse educators provide comprehensive screening assessments and provide ambulatory insulin stabilisation service. GP clincal fellows review and extend the assessments and develop patient-specific management plans, that are discussed and applied with specialist and patient. |  |
| First author and year: Batista et al., 2016 | Location: Brazil, urban, one site | Design and intervention period: Protocol for randomised, controlled study, 12 months | N= 264 Health condition: Coronary artery disease (long-term) | N(HCPs)= not reported | Organisation of care: Use of telemedicine to support the counter-referral of stable CAD patients treated in a tertiary outpatient clinic to primary care facilities, with remote assistance for routine follow-up and decision-making regarding the need for care at the tertiary clinic | No outcomes yet. |
|  |  |  |  |  | Role-distribution: GP provides patient care and discusses it regularly with specialist via tele tool. Specialist provides support when needed and has access to patients' electronic health record. |  |
| First author and year: Bekelman et al., 2013, 2014, 2018 | Location: United States, urban, multiple sites | Design and intervention period: Randomised, controlled study, 36 months | N= 314 Health condition: Heart failure (long-term) | N(HCPs)= not reported | Organisation of care: Evidence-based, algorithm-guided management of breathlessness, fatigue, pain and depression. Regular collaborative team meetings to discuss care and regular follow-up visits via phone with patients. | Heart failure–specific health status (/), depressive symptoms and fatigue (+) pain and shortness of breath (/). |
|  |  |  |  |  | Role-distribution: Nurse performs patient assessment, develops management plan. Social worker performs psychosocial assessment and management. GP and specialist are part of collaborative care team and support in case review and supervise. |  |
| First author and year: Carron et al., 2017 | Location: Switzerland, rural, multiple sites | Design and intervention period: Controlled, before-and-after study, 12 months | N= 57 Health condition: COPD (long-term) | N(GPs)= 3 N(specialists)= not reported | Organisation of care: Six weekly group-based self-management education sessions for patients. Proactive phone calls to remind patients to regularly visit GP for follow-up. Programme coordinators transfer information between HCPs to enhance collaboration and care coordination. | Social and emotional dimensions of health-related quality of life (+), self-efficacy (+), exercise capacity (+), COPD knowledge (+), breathing technique skills (+). |
|  |  |  |  |  | Role-distribution: Pulmonologists, respiratory physiotherapists, specialised nurses and pharmacists provide patient education and self-management sessions. GPs are in charge of patient care. |  |
| First author and year: Connolly et al., 2018 | Location: New Zealand, region (urban and rural), multiple sites | Design and intervention period: Randomised, controlled study, 36 months | N= 1.227 Health condition: long-term care | N(LTC homes)= 63  N(specialists)= not reported | Organisation of care: Development of a facility care plan. Clinical coaching for staff and regular multidisciplinary team meetings. | Avoidable ED admissions of high-risk residents (+) |
|  |  |  |  |  | Role-distribution: GPs and specialists are part of the multidisciplinary team meetings and discuss patient cases. Nurses and caregivers receive clinical coaching. |  |
| First author and year: Falces et al., 2011 | Location: Spain, urban, multiple sites | Design and intervention period: Controlled, before-and-after study, 24 months | N= 3.194 Health condition: Cardiologic diagnoses (long-term) | N(primary care centres)= 7 N(specialists)= not reported | Organisation of care: Integration of a cardiologist into the primary care team. Shared electronic medical records, weekly consultation sessions with specific communication tools (phone, e-mail and webpage). Agreed patient pathways, follow-up processes after discharge and clinical guidelines. Medical education with theoretical and practical training and shared care course. | GP satisfaction (+), information, problem resolution, and communication (+), access primary care to the cardiologist (+).  Control of cholesterol (+), statin treatment (+), control of blood pressure (+), documentation of ejection fraction (+), medical treatment (+). Resources (/) |
|  |  |  |  |  | Role-distribution: Cardiologist travels to GP practice for weekly consultation sessions. Coordination of follow-up and patient care together with GP via new communication tools. |  |
| First author and year: Fortin et al., 2013, 2016 | Location: Canada, region (urban and rural), multiple sites | Design and intervention period: 1) randomised study with delayed intervention arm, 2) before-and-after study, 3) controlled study, 12 months | N= 318 Health condition: Diabetes, cardiovascular disease, COPD, asthma or certain risk factors (long-term) | N(primary care practices)= 8 N(specialists)= not reported | Organisation of care: Patient needs assessment to adapt or develop existing services towards behavioural interventions with focus on self-management support, patient-centred care, and motivational interviewing. Medical teaching for practitioners. | Health Education Impact Questionnaire (HEIQ) in the following domains: health-directed behaviour, emotional well-being, self-monitoring and insight, constructive attitudes and approaches, skill and technique acquisition (++). Prevalence of recommended physical activity (/) BMI results (++). |
|  |  |  |  |  | Role-distribution: Intervention will be carried out upon GP referral. Documentation of exchanges and long-term follow up. Specialists have regular contact and support. Trained nurses performed preliminary clinical evaluation and designed individualised intervention plan. |  |
| First author and year: Haley et al., 2014 | Location: United States, urban, multiple sites | Design and intervention period: Before-and-after study, 15 months | N=  Health condition: chronic kidney disease (at diagnosis and long-term) | N(GP practices)= 9  N(nephrology practices)= 5 | Organisation of care: Improved communication and referral patterns between nephrologists and GPs with educational material, screening protocols, referring clinician faxback form, post-consult letters and patient education diary. | Documentation of glomerular filtration rate (++), GP self-reported awareness of risk factors, the need to track high-risk patients, and the importance of early referral (+), specialist self-reported attention to communication and co-management with primary GP (+), satisfaction among all HCPs (+) |
|  |  |  |  |  | Role-distribution: GP and specialist each develop tools to enhance communication and specify referral processes from their side. Develop and implement co-management plans. |  |
| First author and year: Ho et al., 2018 | Location: Canada, region (mainly urban), multiple sites | Design and intervention period: Protocol for randomised controlled feasibility study, 12 months | N(estimated)= 628 Health condition: geriatric patients (long-term) | N(LCT homes)= 4 N(specialists)= not reported | Organisation of care: Technology-based geriatric pharmacology consultation and review service. Provision of educational material to referring GP and opportunity to review medication and consult. | No outcomes yet. |
|  |  |  |  |  | Role-distribution: GPs provide patient care with support. Specialists support via videoconference telemedicine and in-person consultations. Project staff performs medication review, answer geriatric drug information questions, assess effectiveness of recommendations and monitor adherence. |  |
| First author and year: Martins et al., 2016 | Location: Brazil, urban, multiple sites | Design and intervention period: Before-and-after study, 24 months | N= 360 Health condition: Asthma, COPD (at diagnosis, long-term) | N(GPs, family physicians, pediatricians)= 56 N(pulmonologists)= 4 N(nurses)= 47 N(pharmacists, chest therapists)= 29 | Organisation of care: Educational intervention to improve knowledge (raise awareness and patient case discussions) and collaboration among health care workers by reinforcing the need to build a network. Joint patient consultations, round-table discussions and contacts by phone or email between primary care teams and pulmonologists allowed on-going support, as GPs became more experienced. | Number of referrals to secondary pulmonology care for asthma, COPD and other lung diseases (++), dispensing of beclomethasone propionate inhaler canisters in the 5 months after program start (+). |
|  |  |  |  |  | Role-distribution: GPs and primary care staff attend educational workshops by specialists. GPs and specialists perform joint consultations with patient and discuss afterwards without patients. |  |
| First author and year: Mata-Roman et al., 2013 | Location: Spain, health area (urban and rural), multiple sites | Design and intervention period: Controlled study, 24 months (in two one-year periods) | N= 208 Health condition: gastrointestinal symptoms (at diagnosis) | N(GPs)= 28 N(specialists)= not reported | Organisation of care: Training type intervention with regular meetings to perform joint consultations and clinical sessions on patients. | Referrals to first consultation (+) |
|  |  |  |  |  | Role-distribution: GPs presented their patient cases in the regular meetings to discuss. Specialists take lead in the joint consultations with GPs. |  |
| First author and year: O'Leary et al, 2018 | Location: Australia, health area (urban), multiple sites | Design and intervention period: Controlled, before-and-after study, 12 months | N= 41 Health condition: chronic hepatitis B (long-term) | N(GPs)= 8  N(specialists)= not reported | Organisation of care: Primary care staff receives education sessions regarding diagnosis and management of CHB by specialists and training in the use of the "B in IT" web-based tool was provided. Web-based tool is used for documentation (treatment protocols) and timely communication between HCPs. | Higher overall patients' compliance in intervention then in the control group. |
|  |  |  |  |  | Role-distribution: GPs monitor and treat patients in the community, with continued oversight by hepatologists and clinical nurse consultants. Communicate within the electronic treatment protocol with specialist. Following a GP referral, specialists will determine the patient's CHB phase of disease and the need for an oral antiviral therapy. The patient is referred back to the GP. Inform GPs when a patient should be referred back to the specialist liver clinic. |  |
| First author and year: Ong et al., 2019 | Location: Canada, region (urban and rural), multiple sites | Design and intervention period: Controlled study, 12 months | N= not reported, but 250 traditional referrals and 106 eConsults Health condition: kidney disease (at diagnosis, long-term) | N(GPs)= 52 N(specialists)= 23 | Organisation of care: Web-based eConsult platform provides secure bidirectional communication for physicians between GPs and specialists or between specialists, allowing clinical questions to be asked and supporting documents uploaded. Prior to the launch, GPs were engaged through group meetings to determine top nephrology cases and issues they faced that could be incorporated into the eConsult model. A central triage with a designated clinical case assigner was integrated into the eConsult model to assign cases. | GP self-reported quality of care by timely response from nephrologists (+), nephrologists self-reported productivity (+) |
|  |  |  |  |  | Role-distribution: GPs upload questions and material on the platform for the specialists to answer. |  |
| First author and year: Pang et al., 2016 | Location: Canada, province (urban and rural), multiple sites | Design and intervention period: Before-and-after study, 12 months | N= none Health condition: chronic kidney disease (at diagnosis) | N(GPs)= 68 N(specialists)= 7 N(nurses)= not reported | Organisation of care: GPs were designated as mentees and nephrologists were designated as mentors. Training sessions on role-distribution within the project and medical education for GPs on nephrologic care. Regular regional group learning sessions and case consultations via telephone. | On average, active mentees participated in 2 one-on-one consultations. Consultations most commonly occurred via e-mail (88%) and were often resolved without in-person follow-up.  Self-rated knowledge of and comfort levels with taught topics (++), mean satisfaction scores for all measured modes of communication (+). |
|  |  |  |  |  | Role-distribution: GPs receive training by specialists, attend regular learning sessions and initiate case discussions. Specialists provide support as a mentor. Nurse practitioners and registered nurses share their roles with GP. |  |
| First author and year: Porter et al., 2015/ Hynes et al., 2017/ Hynes et al., 2019 | Location: United States, urban, one site | Design and intervention period: Before-and-after study, 24 months | N= 175 Health condition: end-stage renal disease (long-term) | N(GPs)= not reported N(specialists)= 8 N(nurse coordinator, pharmacists, community health worker)= not reported | Organisation of care: The intervention expands the existing care team of the dialysis unit (comprised of a nephrologist, dialysis nurse/ nurse manager, dialysis technician, social worker, and dietitian) to include a nurse coordinator, general internist, pharmacist, and CHW, all of whom will see the patient during dialysis treatments and separately as needed. | HRQoL assessment: Physical Composite Scale from baseline to 6 months (++), Mental Composite Scale from baseline to 12 and 18 months (++), Burden domain from baseline to 18 months (+), symptoms domain from baseline to 6 months (++), but from baseline to 12 months (+), effects domain from baseline to 6, 12, and 18 months (++). |
|  |  |  |  |  | Role-distribution: Specialists and GPs were co-lead in the multidisciplinary teams. All HCP perform rounds on patients monthly and review patients in team meetings. GPs conduct initial patient intakes, perform follow-up visits as needed, document in the electronic medical record and communicate with other HCPs vial flow sheets, email and consultations. Specialists provide care for comorbid conditions and preventive care. Nurse coordinator monitors episodic inpatient care and schedules assessments or procedures with other HCPs. Pharmacists conduct medication assessment, support medication compliance and monitor immunisations. Community health workers serve as a liaison between community, patient/family and care team. |  |
| First author and year: Santoro et al., 2019 | Location: Italy, region (urban and rural), multiple sites | Design and intervention period: Before-and-after study, 156 months | N= 25.257 Health condition: chronic kidney disease (at diagnosis, long-term) | N(HCPs)= not reported | Organisation of care: Teaching seminars to improve early identification of the population at risk, to promote effective prevention strategies (lifestyle modification, correction of modifiable risk factors) and treatments, and to avoid inappropriate drug utilisation. Implementation phase consisted in opening specialised outpatient facilities managed by GPs. Co-management between the GP and the nephrologist is maintained through the use of a dedicated website and an e-mail account that permanently connect the GP to the nephrological team. | Mean glomerular filtration rate (+) |
|  |  |  |  |  | Role-distribution: Roles in the project are distributed according to the patient's stage of CKD. The higher the stage of CKD, the more involved the specialist becomes and the less tasks are distributed to the GP. Both GPs and specialists perform clinical assessments to diagnose disease, as well as management and lifestyle modifications and action plans. |  |
| First author and year: Steurer-Stey et al., 2014/ Markun et al., 2018 | Location: Switzerland, urban, multiple sites | Design and intervention period: Randomised, controlled trial, 12 months | N= 216 Health condition: COPD ( at diagnosis) | N(GPs)= 33 N(specialists)= not reported | Organisation of care: Training workshop provided by pneumologist and respiratory physiotherapist for GPs on how to provide COPD care. Specialists develop a checklist from existing guideline as support for GPs. | Mean composite score of implemented key elements in intervention group (+) |
|  |  |  |  |  | Role-distribution: GPs receive training at baseline and after 6months and are encouraged to use provided checklist. Specialists provide information and experience exchange in educational workshop. |  |
| First author and year: Scherpbier-de Haan et al., 2013 | Location: Netherlands, urban academic network, one site | Design and intervention period: Randomised, controlled trial, 12 months | N= 164 Health condition: hypertension or diabetes type 2 (long-term) | N(GP practices)= 9 N(specialists)= not reported N(nurse practitioners)= not reported | Organisation of care: Shared care model between GP, nurse practitioner and specialist. A nephrology team teaches primary care staff and provide a protocol based on guideline on treatment goals and advice. | Systolic blood pressure (+), diastolic blood pressure (/) between intervention and control group.  Blood pressure after 1 year (+), cholesterol and low-density lipoprotein levels, use of lipid-lowering drugs (+). |
|  |  |  |  |  | Role-distribution: Specialists educate GPs and nurse practitioners on a regular basis. Nurse practitioners are in charge of patient care and GPs oversee care provided. Specialists support with information on digital platform and answer questions. |  |
| First author and year: Tinetti et al., 2016/ Blaum et al., 2018 | Location: United States, state, multiple sites | Design and intervention period: Before-and-after study, 10 months | N= 119 Health condition: 3 or more chronic conditions (long-term) | N(GPs)= 9 N(specialists)= 5 N(other HCPs)= not reported | Organisation of care: Patient priority-directed care is developed by stakeholders and primary and secondary care are then aligned. All members of the team, including patients and caregivers, must be willing and able to carry out their agreed-on roles and responsibilities, which are determined by the patient’s conditions, outcome goals, and care preferences. All clinicians work form an integrated plan share information and care. | No outcomes yet. |
|  |  |  |  |  | Role-distribution: GPs and specialists receive trainings sessions on communication and medical education. They participate in collaborative learning sessions and review selected patient cases, share care and information. |  |
| First author and year: van Gelder et al., 2017 | Location: Netherlands, country, multiple sites | Design and intervention period: Randomised controlled trial, 12 months | N= 3.004 Health condition: chronic kidney disease (long-term) | N(GPs)= 128 N(specialists)= not reported | Organisation of care: A web-based consultation platform as an add-on in the existing electronic referral system. Telenephrology accessed individual patient data from the EMR only after the GP opened the consultation module for that specific patient. The specialist has access to patient data then, is notified about the consultation and advices the GP on how to treat and when to refer. | In comparison to the baseline results, registration of eGFR on the EMR episode list increased by an absolute 14.9% in the intervention group and by 16.4% in the control group, mean costs per patient (-) |
|  |  |  |  |  | Role-distribution: GPs consult with nephrologist and if not sufficient, additional information or questions can be directed at the specialist. Specialist advices on care and referral processes. |  |
