# Supplementary material for: Improvement Strategies for the Challenging Collaboration of General Practitioners and Specialists for Patients with Complex Chronic Conditions: A Scoping Review
Source: Int J Integr Care. 2022 Aug 8;22(3):4. doi: 10.5334/ijic.5970 (PMC9374013; doi:10.5334/ijic.5970)
Supplement: Supporting Tables. — Tables 1 to 3. [file ijic-22-3-5970-s1.zip › s1-ijic-5970_tomaschek/5970-26308-1-SP.docx]

Supporting Table 1: PICO format for generation of research question

| Population | patients with complex chronic disease requiring long-term care |
| --- | --- |
| Intervention | collaboration by medical specialists and general practitioners |
| Comparison | usual care, other care models |
| Outcome | concepts and components of the interface for collaboration, role-distributions between medical specialists and general practitioners |
