# Supplementary material for: Improvement Strategies for the Challenging Collaboration of General Practitioners and Specialists for Patients with Complex Chronic Conditions: A Scoping Review
Source: Int J Integr Care. 2022 Aug 8;22(3):4. doi: 10.5334/ijic.5970 (PMC9374013; doi:10.5334/ijic.5970)
Supplement: Supporting Tables. — Tables 1 to 3. [file ijic-22-3-5970-s1.zip › s1-ijic-5970_tomaschek/5970-26309-1-SP.docx]

Supporting Table 2: Detailed search string for PubMed database

| **Description** | **Details** |
| --- | --- |
| Patient population  Exclusion of children | ((complex[Title/Abstract] OR "complex patients"[Title/Abstract] OR "complex patient"[Title/Abstract] OR "medical complex"[Title/Abstract] OR "medically complex"[Title/Abstract] OR "complex health"[Title/Abstract] OR "complex health needs"[Title/Abstract] OR "complex needs"[Title/Abstract] OR "health services needs and demand"[MeSH] OR "chronic disease"[MeSH] OR ("chronic"[Title/Abstract] AND "disease"[Title/Abstract]) OR "chronic disease"[Title/Abstract] OR "chronic"[Title/Abstract] OR "chronic"[Title/Abstract] AND illness[Title/Abstract] OR "chronic illness"[Title/Abstract] OR ("chronic"[Title/Abstract] AND conditions[Title/Abstract]) OR "chronic conditions"[Title/Abstract] OR "chronic disease"[MeSH] OR ("chronic"[Title/Abstract] AND "disease"[Title/Abstract] ) OR "chronic disease"[Title/Abstract] OR ("chronic"[Title/Abstract] AND "diseases"[Title/Abstract]) OR "chronic diseases"[Title/Abstract] OR (chronic[Title/Abstract] AND "health conditions"[Title/Abstract]) OR "health condition"[Title/Abstract] OR "multimorbidity"[MeSH])  NOT ("pediatrics"[MeSH] OR "pediatrics"[Title/Abstract] OR "paediatric"[Title/Abstract] OR juvenile[Title/Abstract] OR "child"[MeSH] OR "child"[Title/Abstract] OR "children"[Title/Abstract] OR "infant"[MeSH] OR "infant"[Title/Abstract] OR “infants"[Title/Abstract]) |
| General practitioners  Exclusion of other primary care professions | AND ("primary health care"[MeSH] OR "physicians, primary care"[MeSH] OR ("physicians"[Title/Abstract] AND "primary care"[Title/Abstract]) OR "primary care physicians"[Title/Abstract] OR "primary care physician"[Title/Abstract] OR "primary health care"[Title/Abstract] OR "physicians"[MeSH] OR "physicians"[Title/Abstract] OR "doctor"[Title/Abstract] OR "general practitioners"[MeSH] OR ("general"[Title/Abstract] AND "practitioners"[Title/Abstract]) OR "general practitioners"[Title/Abstract] OR ("general"[Title/Abstract] AND "practitioner"[Title/Abstract]) OR "general practitioner"[Title/Abstract] OR "general practitioners"[MeSH] OR "community"[Title/Abstract] OR "community health services"[MeSH])  NOT (“nurse”[Title] or “nurses” [Title] or “nursing”[Title] or “nurse practitioners”[Title] or “nurse practitioners”[Title] “nurse-led”[Title] or “pharmacist”[Title] or “pharmacists”[Title] or “pharmacies”[Title] or “pharmacy”[Title] or “therapist”[Title] or “therapists”[Title]) |
| Specialists | AND ("physicians"[MeSH] OR "secondary care"[MeSH] OR "cardiologists"[MeSH] OR “cardiologists"[Title/Abstract] OR "cardiologist"[Title/Abstract] OR "dermatologists"[MeSH] OR "dermatologists"[Title/Abstract] OR "dermatologist"[Title/Abstract] OR "endocrinologists"[MeSH] OR "endocrinologists"[Title/Abstract] OR "endocrinologist"[Title/Abstract] OR "hospitalists"[MeSH] OR "hospitalists"[Title/Abstract] OR "hospitalist"[Title/Abstract] OR "nephrologists"[MeSH] OR "nephrologists"[Title/Abstract] OR "nephrologist"[Title/Abstract] OR "neurologists"[MeSH] OR "neurologists"[Title/Abstract] OR "neurologist"[Title/Abstract] OR "gastroenterologists"[MeSH] OR "gastroenterologists"[Title/Abstract] OR "gastroenterologist"[Title/Abstract] OR "oncologists"[MeSH] OR "oncologists"[Title/Abstract] OR "oncologist"[Title/Abstract] OR"ophthalmologists"[MeSH] OR "ophthalmologists"[Title/Abstract] OR "ophthalmologist"[Title/Abstract] OR "pulmonologists"[MeSH] OR "pulmonologists"[Title/Abstract] OR "pulmonologist"[Title/Abstract] OR "radiologists"[MeSH] OR "radiologists"[Title/Abstract] OR "radiologist"[Title/Abstract] OR "rheumatologists"[MeSH] OR "rheumatologists"[Title/Abstract] OR "rheumatologist"[Title/Abstract] OR "surgeons"[MeSH] OR "surgeons"[Title/Abstract] OR "surgeon"[Title/Abstract] OR "urologists"[MeSH] OR "urologists"[Title/Abstract] OR "urologist"[Title/Abstract] OR “hospital”[Title/Abstract] OR “hospitals”[Title/Abstract] OR "specialization"[MeSH] OR "specialization"[MeSH Terms] OR "specialization"[Title/Abstract] OR "specialist"[Title/Abstract] OR "specialists"[Title/Abstract]) |
| Type of care provided | AND ("intersectoral collaboration"[MeSH] OR “interorganizational”[Title/Abstract] OR "primary-secondary"[Title/Abstract] OR “collaboration”[Title/Abstract] OR “collaborating”[Title/Abstract] OR “collaborative”[Title/Abstract] OR “collective”[Title/Abstract] OR "joint"[Title/Abstract] OR “shared”[Title/Abstract] OR “sharing”[Title/Abstract] OR "integration"[Title/Abstract] OR “integrated”[Title/Abstract] OR “integrating”[Title/Abstract] OR “cooperation”[Title/Abstract] OR “cooperating”[Title/Abstract] OR “cooperative”[Title/Abstract] OR “team”[Title/Abstract] OR "continuity of patient care"[MeSH] OR "long-term care"[MeSH] OR "delivery of health care, integrated"[MeSH]) |
| Exclusion of non-eligible study design | NOT (“qualitative”[Title] OR “focus group”[Title] OR “case report”[Title] OR “surgery”[Title] OR “transplantation”[Title] OR “invasive”[Title] OR “non-invasive”[Title] OR “noninvasive”[Title] OR “shared decision-making”[Title] OR “shared decision making”[Title] OR “self-care”[Title] OR “self-management”[Title] OR “cost”[Title] OR “economic”[Title] OR “reimbursement”[Title] OR “prevalence”[Title] OR “incidence”[Title] OR “tooth”[Title] OR “teeth”[Title] OR “dentist”[Title])) |
| Publication period | AND "2010/03/04"[PDAT] : "2020/03/04"[PDAT] |
